# Indolent T-cell Lymphoproliferative Disorder of the Gastrointestinal Tract Mimicking Crohn’s Disease

**DOI:** 10.7759/cureus.60467

**Published:** 2024-05-16

**Authors:** Jesus Delgado-de la Mora, Daniel Montante-Montes de Oca, Arturo Ángeles-Ángeles, Leticia Quintanilla de Fend, Braulio Martínez Benitez

**Affiliations:** 1 Pathology, Weill-Cornell Medicine, New York City, USA; 2 Pathology, Instituto Nacional de Ciencias Médicas y Nutrición Salvador Zubirán, Mexico City, MEX; 3 Faculty of Medicine, University of Tübingen, Tübingen, DEU

**Keywords:** ulcerative colitis, crohn's disease, indolent t-cell lymphoma, inflammatory bowel disease, intestinal t-cell lymphoma

## Abstract

Indolent clonal T-cell lymphoproliferative disorder (iCTLD-GI)/indolent T-cell lymphoma of the gastrointestinal tract (iTLP-GI) poses diagnostic challenges, and despite its rarity, accurate diagnosis is crucial for appropriate management. We report the case of 34-year-old female with a 19-year history of gastrointestinal symptoms suggestive of inflammatory bowel disease (IBD). Subsequent evaluation revealed iCTLD-GI/iTLP-GI with extensive Crohn's disease-like morphological alterations, previously unreported. These macroscopic and microscopic aspects underscore the need for a comprehensive evaluation to avoid misdiagnosis with IBD. Additionally, molecular studies have identified potential therapeutic targets, highlighting the evolving management strategies. This case underscores the diagnostic complexity of iCTLD-GI/iTLP-GI, especially when the condition mimicks IBD such as Crohn's disease.

## Introduction

Indolent clonal T-cell lymphoproliferative disorder (iCTLD-GI) [1] or indolent T-cell lymphoma of the gastrointestinal tract (iTLP-GI) [2] is a rare entity. Since its original description in 1994 [3], less than 70 cases have been reported worldwide [4]. Its diagnosis can be challenging, especially in biopsies or when it involves multiple sites outside the gastrointestinal tract (GI), requiring the integration of clinical data, morphology, endoscopy, immunohistochemistry studies and preferably evaluation of T cell receptor (TCR) rearrangements.

Unlike other primary lymphomas of the gastrointestinal tract such as enteropathy-associated T-cell lymphoma or monomorphic epitheliotropic intestinal T-cell lymphoma, which are characterized by aggressive behavior with an average survival of less than 10 months [5,6], iCTLD-GI/iTLP-GI has a better prognosis with survival extending to more than 10 years [7]; however, patients frequently present significant morbidity, debilitating symptoms, and a risk of transformation to high-grade lymphoma, documented in around 10% of the cases [4]. 

Due to the long evolution of this entity, the identification of morphological alterations in the GI such as ulcers, glandular distortion, or granulomas secondary to their rupture is not uncommon, which can simulate and lead to its misdiagnosis as inflammatory bowel disease (IBD) [4,8-10]. There are multiple reports of iCTLD-GI/iTLP-GI with a previous diagnosis of IBD [4,8-10]; however, in the cases in which it was possible to evaluate the material where this diagnosis was made, it was identified that these were morphological characteristics that simulated IBD in patients with iCTLD-GI/iTLP-GI [4,9].

The importance of correctly identifying this entity lies in the fact that the current therapeutic approach for patients with iCTLD-GI/iTLP-GI is conservative [4,8]. Therefore, avoiding diagnostic confusion with T-cell lymphomas or IBD would avoid unnecessary or excessively aggressive treatments. In this sense, we present the case of a patient with a 19-year clinical history of symptoms and with extensive Crohn’s disease (CD)-like morphological changes, previously undescribed.

## Case presentation

A 34-year-old female patient had an initial onset of her condition 19 years earlier with abdominal pain, vomiting, stools with reduced consistency, and a loss of 29 kg in seven years of evolution. During this time, she underwent two colonoscopy investigations with diagnoses of spastic colon and eosinophilic inflammation, receiving pharmacological treatment without improvement.

She arrived at our institution with a clinical picture of 13 years of evolution, at the age of 28. Initially, the possibility of celiac disease was ruled out with negative HLA DQ2/DQ8 antigen, anti-endomysial and anti-transglutaminase immunoglobulin A (IgA) antibodies; duodenal biopsies showed no alterations, without villous atrophy or glandular hyperplasia and with less than 10 lymphocytes per 100 enterocytes. A computed axial tomography (CT) scan was requested where mesenteric lymphadenopathy, decreased caliber and concentric mural thickening were observed in the fourth portion of the duodenum and in segments of the jejunum and ileum; no alterations were observed in the colon.

**Figure 1:**
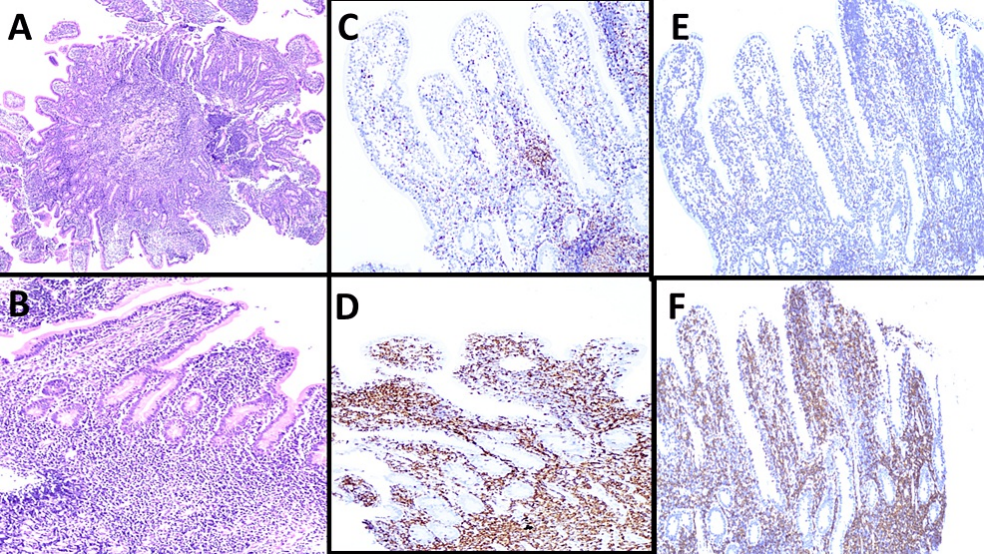
Microscopic findings of jejunum biopsy (A,B) Microscopic examination (hematoxylin & eosin stain) of jejunum biopsy with infiltration by iCTLD-GI/iTLP-GI (A, 4x), composed of a monomorphic population with scarce atypia, without glandular destruction or epitheliotropism (B, 10x). Immunohistochemistry reactions (10x) with reactive positivity for CD20 (C) in B lymphocytes, neoplastic cells positive for CD3 (D), negative for CD4 (E) and positive for CD8 (F).

Subsequently, antegrade double ballon enteroscopy was performed, where nodular mucosa was observed in the duodenum and jejunum, for which biopsies were taken. In both sites, infiltration by a lymphoid population extending from the lamina propria to the submucosa was identified, generating villous thickening. It was composed of medium-sized cells with scarce atypia, without damage to the glandular or vascular component and without an increase in intraepithelial lymphocytes (<20/100 epithelial cells) (Figure 1A-1B). This population was positive for CD3, CD8, CD7, TIA-1, and had a Ki-67 <10%, it was negative for CD20, CD4, CD56, EBER, and CD103 (Figure 1C-1F). Cytogenetic studies showed no abnormalities (46XX).

With all the above, the diagnosis of iCTLD-GI/iTLP-GI was made, and treatment initiated with oral methotrexate, 10 mg per week. A CT scan was performed seven months after the diagnosis, identifying the persistence of dilation and diffuse mural thickening of different segments of the small intestine, splenomegaly of 14 cm in the long axis, and mesenteric lymphadenopathy of up to 12 mm.

**Figure 2:**
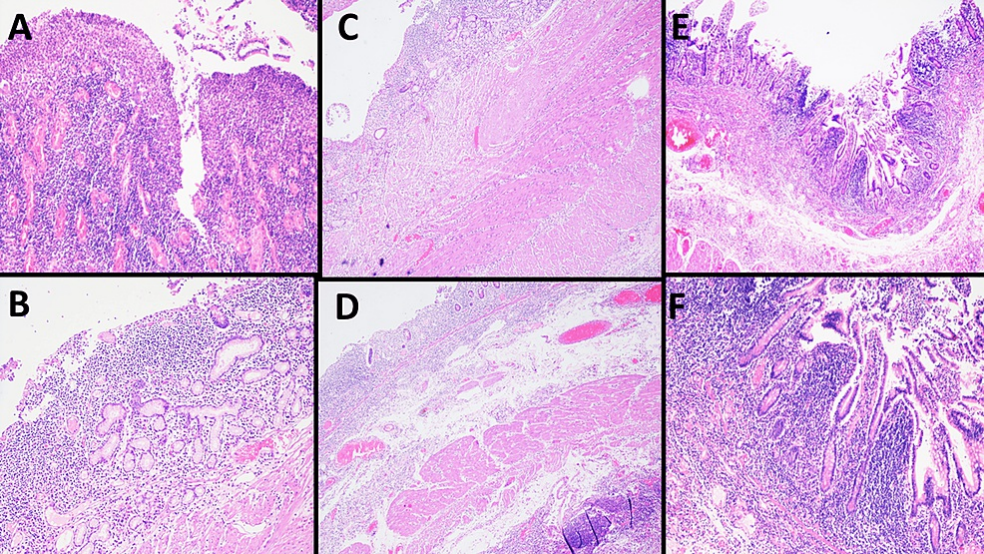
Microscopic findings of the segments of the small bowel. Microscopic examination (hematoxylin & eosin stain) of the segments of small bowel with the presence of fissures that extend to the muscularis mucosa (A, 10x), pseudopyloric metaplasia (B, 10x), thickening of the muscularis mucosae and muscularis propria (C, 4x), transmural lymphoid aggregates (D, 4x) and infiltration by iCTLD-GI/iTLP-GI (E, 4x) with extension to the submucosa (F, 10x).

One year after her last consultation, the patient presented with intestinal obstruction with subsequent resection of a segment of small intestine in a hospital in her hometown. We subsequently received blocks with paraffin-embedded tissue from this surgical specimen, in which we discontinuously identified infiltration by iCTLD-GI/iTLP-GI with the same morphological and immunohistochemical characteristics previously described, the disease extending to the submucosa without having contact with the muscularis propria. Additionally, we observed multiple CD-like morphological alterations, such as mucosa with erosions, fissures, glands with bifurcations, pseudopyloric metaplasia, transmural lymphoid aggregates (CD20+) and thickening of the muscularis propria (Figure 2A-2F).

**Figure 3:**
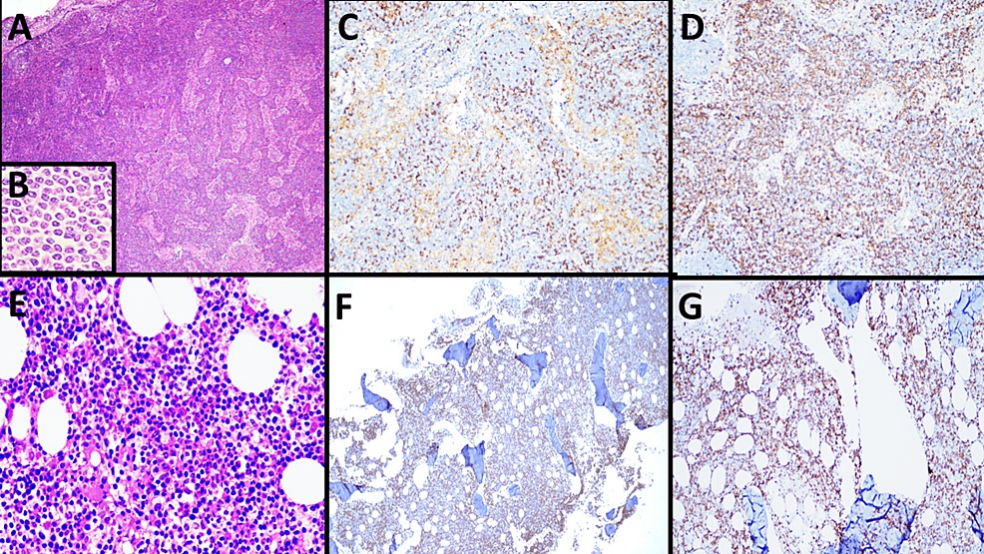
Microscopic findings of mesenteric lymph node and bone marrow biopsies Microscopic examination (hematoxylin & eosin stain) of mesenteric lymph node with paracortical infiltration by iCTLD-GI/iTLP-GI (A, 4x) composed of monomorphic population with scarce atypia (B, 40x), immunohistochemistry reactions with focal positivity for CD3 in neoplastic cells (C, 10x) and diffuse for CD4 (D, 10x). Microscopic examination (hematoxylin & eosin stain) of bone marrow with interstitial infiltration by iCTLD-GI/iTLP-GI with the same cytological characteristics previously described (E, 10x), immunohistochemistry reactions with positivity for CD3 in neoplastic cells (F, 4x) and for CD4 (G, 10x).

Dissemination of the disease to mesenteric lymph nodes with a paracortical pattern (Figure 3A-3D) and to bone marrow with an interstitial pattern (Figure 3E-3G) was noted. The cytological and immunophenotypic appearance was the same as previously described. DNA extraction was carried out from paraffin blocks of the small intestine corresponding to the intestinal occlusion event. Subsequently, a polymerase chain reaction was performed in search of TCR rearrangements based on the BIOMED-2 protocol [11], in which a monoclonal population for TCR-β was identified.

Nineteen years since the beginning of her symptoms and five years since her diagnosis, the patient is currently still under observation, asymptomatic and being treated with methotrexate. On follow-up CT scan, the dilation of the small intestine persists in the patient, with mural thickening in the ileum, mesenteric/retroperitoneal lymphadenopathy, and splenomegaly.

## Discussion

iCTLD-GI/iTLP-GI is a rare entity, characterized by a clonal lymphoid population of T lineage with scarce atypia, monomorphic, without glandular destruction or epitheliotropism, with a low proliferation index (Ki-67 <10%), absence of CD56 expression and not associated with Epstein-Barr virus [1,4]. Despite having reported survivals of more than a decade [7], patients frequently present debilitating events such as vomiting, diarrhea, intestinal obstructions or perforations [3-4,7-10,12,13]; likewise, cases of transformation to high-grade T-cell lymphomas with fatal results have been documented [3-4,9,10,13].

Despite all the previously described characteristics, the differential diagnosis with entities such as hepatosplenic T-cell lymphoma and IBD can be challenging in cases with scarce tissue (biopsies), disease disseminated to sites outside the GI or with morphological changes that mimic ulcerative colitis (UC)/CD [4,8-10,14].

In this sense, multiple cases of iCTLD-GI/iTLP-GI with a previous diagnosis of UC or CD have been described [4, 8-10]; these diagnoses have been supported by morphological aspects such as ulcers, alteration of glandular architecture or granulomas secondary to their rupture [4, 8-10]; although these morphological changes can be found in IBD, they are not exclusive to these entities, documenting the same and some additional ones such as fissures, pseudopyloric metaplasia, transmural lymphoid aggregates and thickening of the muscular layer as reparative phenomena and a ubiquitous response to damage [15-18], so it is plausible that they may be present in cases of iCTLD-GI/iTLP-GI, as it is a chronic disease that involves the mucosa.

Given the presence of morphological alterations that simulate IBD in patients with iCTLD-GI/iTLP-GI, it has previously been proposed that although this case may present alterations such as ulcers and glandular bifurcation or granulomas, they are not usually accompanied by additional morphological alterations found in UC or CD, this being key to its differentiation [8]; however, this case documents CD-like morphological findings, previously not described in iCTLD-GI/iTLP-GI, such as fissures, pseudopyloric metaplasia, muscularis propria thickening and transmural lymphoid aggregates.

The above is previously relevant given that at least ~14% of published iCTLD-GI/iTLP-GI cases to date have had a prior diagnosis of IBD [4, 8-10], and in all the cases in which it was possible to reevaluate the material supporting this diagnosis, it was identified that it was iCTLD-GI/iTLP-GI with morphological changes that simulate IBD, taking an average of 8.6 years until its correct identification [4,9] (Table 1). Although this entity is not frequent, it is striking that within the 70 published cases, no alterations such as those found in this patient were described, which suggests that some of these cases could be underdiagnosed as IBD.

**Table 1:** Cases of iCTCLD-GI/iTCL-GI with a previous diagnosis of inflammatory bowel disease iCTCLD-GI/iTCL-GI: indolent clonal T-cell lymphoproliferative disorder / indolent T-cell lymphoma of gastrointestinal tract; AWD: alive with disease; AWOD: alive without disease; DWDP: died because disease progression; F: female; M: male; UC: ulcerative colitis; CD: Crohn's disease; IBD: inflammatory bowel disease.

Case	Study	Age/Sex	Presumptive diagnosis	Time until diagnosis of ICTCLDGI/ITCLGI	Sites of involvement	Reevaluation of IBD diagnosis	Follow-up	Outcome
1	Perry et al. 2013 [8]	15/F	UC	3 years	Jejunum, ileum, colon	No	52 months	AWD
2	Perry et al. 2013 [8]	46/M	CD	Not specified	Oral cavity, ileum, colon	No	9 months	AWD
3	Perry et al. 2013 [8]	50/M	IBD	7 years	Duodenum	No	84 months	AWOD
4	Perry et al. 2013 [8]	77/F	CD	Not specified	Oral cavity, ileum	No	168 months	AWD
5	Perry et al. 2019 [9]	37/M	CD	8 years	Oral cavity, ileum, colon	Reevaluated and iCTCLD-GI/iTCL-GI	96 months	DWDP
6	Soderquist et al. 2020 [10]	49/M	CD	5 years	Duodenum, jejunum	No	228 months	AWD
7	Fan et al. 2023 [4]	51/M	UC	4 years	Oral cavity, ileum, colon, rectum	Reevaluated and iCTCLD-GI/iTCL-GI	54 months	DWDP
8	Fan et al. 2023 [4]	33/M	CD	8 years	Distal ileum, colon, rectum	Reevaluated and iCTCLD-GI/iTCL-GI	38 months	AWD
9	Fan et al. 2023 [4]	41/M	UC	13 years	Ileum, colon	Reevaluated and iCTCLD-GI/iTCL-GI	50 months	AWD
10	Fan et al. 2023 [4]	30/M	UC	10 years	Colon	Reevaluated and iCTCLD-GI/iTCL-GI	6 months	AWD

The implications of a misdiagnosis of IBD in a patient with iCTLD-GI/iTLP-GI may be multiple. In relation to this, the case of a patient with a confirmed diagnosis of CD was previously reported who, after presenting a poor response to treatment (steroids, azathioprine), was started on a tumor necrosis factor-alpha (TNF-α) inhibitor (adalimumab). Seven months later, polypoid lesions were identified in multiple areas of the colon that were diagnosed as iCTLD-GI/iTLP-GI CD8+ with a monoclonal population for TCRγ and TCRβ, the previous biopsies where CD was originally diagnosed did not present monoclonal populations, and later upon the withdrawal of adalimumab, the infiltrate regressed [14].

The previous case suggests a potential effect between TNF-α inhibitors and iCTLD-GI/iTLP-GI CD8+, who could receive this therapy due to a misdiagnosis of IBD. It has previously been documented that TNF-α inhibitors reduce oxidative stress, mitochondrial hyperpolarization and the generation of DNA damage in human CD8+ T lymphocytes, prolonging their life [19], which could influence the persistence and/or dissemination of this entity.

The current treatment suggested in patients with iCTLD-GI/iTLP-GI is conservative and not curative. Due to this, multiple molecular studies in search of potential therapeutic targets have been performed, identifying STAT3-JAK2 gene fusions in four CD4+/CD8- cases [13], alterations in the JAK-STAT signaling pathway in CD4+/CD8- and CD4+/CD8+ [4,10] and in epigenetic-modifying genes such as TET2, DNMT3A and KMT2D in CD4-/CD8+ cases [4,10]. There is uncertainty about a potential association between CD4/CD8 expression and mutational profile.

## Conclusions

In conclusion, we present the case of a patient with iCTLD-GI/iTLP-GI with 19 years of clinical evolution and Crohn’s disease-like morphological changes, some of them previously not reported, and potentially causing underdiagnosis of this entity. Although, without additional data, the morphological appearance may suggest IBD, the integration of the clinical history, the nodular endoscopic appearance, the infiltration by a clonal lymphoid population and potentially the identification of some of the molecular alterations previously described can help support the diagnosis of iCTLD-GI/iTLP-GI.

## References

[1] Campo E, Jaffe ES, Cook JR (2022). The International Consensus Classification of Mature Lymphoid Neoplasms: a report from the Clinical Advisory Committee. Blood.

[2] Bhagat G, Takeuchi K, Naresh KN, Dave SS (2024). Indolent T-cell lymphoma of the gastrointestinal tract. WHO Classification of Tumours Editorial Board. Haematolymphoid Tumours.

[3] Carbonnel F, Lavergne A, Messing B (1994). Extensive small intestinal T‐cell lymphoma of low‐grade malignancy associated with a new chromosomal translocation. Cancer.

[4] Fan W, Niu L, He H (2023). Indolent T-cell lymphoproliferative disorder of gastrointestinal tract with unusual clinical courses: report of 6 cases and literature review. Virchows Arch.

[5] Delabie J, Holte H, Vose JM (2011). Enteropathy-associated T-cell lymphoma: clinical and histological findings from the international peripheral T-cell lymphoma project. Blood.

[6] Tse E, Gill H, Loong F (2012). Type II enteropathy-associated T-cell lymphoma: a multicenter analysis from the Asia Lymphoma Study Group. Am J Hematol.

[7] Margolskee E, Jobanputra V, Lewis SK, Alobeid B, Green PH, Bhagat G (2013). Indolent small intestinal CD4+ T-cell lymphoma is a distinct entity with unique biologic and clinical features. PLoS One.

[8] Perry AM, Warnke RA, Hu Q (2013). Indolent T-cell lymphoproliferative disease of the gastrointestinal tract. Blood.

[9] Perry AM, Bailey NG, Bonnett M, Jaffe ES, Chan WC (2019). Disease progression in a patient with indolent T-cell lymphoproliferative disease of the gastrointestinal tract. Int J Surg Pathol.

[10] Soderquist CR, Patel N, Murty VV (2020). Genetic and phenotypic characterization of indolent T-cell lymphoproliferative disorders of the gastrointestinal tract. Haematologica.

[11] Langerak AW, Groenen PJ, Brüggemann M (2012). EuroClonality/BIOMED-2 guidelines for interpretation and reporting of Ig/TCR clonality testing in suspected lymphoproliferations. Leukemia.

[12] Leventaki V, Manning JT Jr, Luthra R (2014). Indolent peripheral T-cell lymphoma involving the gastrointestinal tract. Hum Pathol.

[13] Sharma A, Oishi N, Boddicker RL (2018). Recurrent STAT3-JAK2 fusions in indolent T-cell lymphoproliferative disorder of the gastrointestinal tract. Blood.

[14] Nascimbeni R, Di Fabio F, Di Betta E, Mariani P, Fisogni S, Villanacci V (2005). Morphology of colorectal lymphoid aggregates in cancer, diverticular and inflammatory bowel diseases. Mod Pathol.

[15] Esaki M, Umeno J, Kitazono T, Matsumoto T (2015). Clinicopathologic features of chronic nonspecific multiple ulcers of the small intestine. Clin J Gastroenterol.

[16] Goldenring JR (2018). Pyloric metaplasia, pseudopyloric metaplasia, ulcer-associated cell lineage and spasmolytic polypeptide-expressing metaplasia: reparative lineages in the gastrointestinal mucosa. J Pathol.

[17] Limantoro I, Lee AF, Rosenbaum DG (2022). Spectrum of bowel wall thickening on ultrasound with pathological correlation in children. Pediatr Radiol.

[18] Edison N, Belhanes-Peled H, Eitan Y (2016). Indolent T-cell lymphoproliferative disease of the gastrointestinal tract after treatment with adalimumab in resistant Crohn's colitis. Hum Pathol.

[19] Otano I, Alvarez M, Minute L (2020). Human CD8 T cells are susceptible to TNF-mediated activation-induced cell death. Theranostics.

